# Angiopoietin–Tie signalling in the cardiovascular and lymphatic systems

**DOI:** 10.1042/CS20160129

**Published:** 2016-12-09

**Authors:** Lauri Eklund, Jaakko Kangas, Pipsa Saharinen

**Affiliations:** * Oulu Center for Cell-Matrix Research, Faculty of Biochemistry and Molecular Medicine, and Biocenter Oulu, Aapistie 5A, FI-90014 Oulu, Finland; † Translational Cancer Biology Program, Research Programs Unit, University of Helsinki, and Wihuri Research Institute, Biomedicum Helsinki, Haartmaninkatu 8, P.O.B. 63, FI-00014 University of Helsinki, Finland

**Keywords:** angiogenesis, angiopoietin, Angpt, Ang1, Ang2, cardiovascular disease, lymphangiogenesis, Tie1, Tie2, TEK, VE-PTP, integrin

## Abstract

Endothelial cells that form the inner layer of blood and lymphatic vessels are important regulators of vascular functions and centrally involved in the pathogenesis of vascular diseases. In addition to the vascular endothelial growth factor (VEGF) receptor pathway, the angiopoietin (Ang)–Tie system is a second endothelial cell specific ligand–receptor signalling system necessary for embryonic cardiovascular and lymphatic development. The Ang–Tie system also regulates postnatal angiogenesis, vessel remodelling, vascular permeability and inflammation to maintain vascular homoeostasis in adult physiology. This system is implicated in numerous diseases where the vasculature has an important contribution, such as cancer, sepsis, diabetes, atherosclerosis and ocular diseases. Furthermore, mutations in the *TIE2* signalling pathway cause defects in vascular morphogenesis, resulting in venous malformations and primary congenital glaucoma. Here, we review recent advances in the understanding of the Ang–Tie signalling system, including cross-talk with the vascular endothelial protein tyrosine phosphatase (VE-PTP) and the integrin cell adhesion receptors, focusing on the Ang–Tie system in vascular development and pathogenesis of vascular diseases.

## INTRODUCTION

Endothelial cells (EC) that form the inner layer of blood and lymphatic vessels are key mediators of vascular functions, including the growth of new blood (angiogenesis) and lymphatic vessels (lymphangiogenesis), tissue fluid homeostasis, vascular permeability and inflammation. Therefore, ECs are centrally involved in numerous diseases, characterized by vessel dysfunction, barrier breakdown and excess or insufficient angiogenesis, such as in diabetes, sepsis, neovascular eye disease, cancer and atherosclerosis. The vascular endothelial growth factor receptor (VEGFR) system is the master regulator of angiogenesis and lymphangiogenesis during vascular development and in neovascularization in adults [1]. The angiopoietin (Ang)–Tie pathway forms the second endothelial growth factor receptor signalling pathway, which performs a necessary function to regulate blood and lymphatic vessel remodelling during midgestation, after the VEGF-VEGFR driven phase of active angiogenenesis. Subsequently, the Ang–Tie system contributes to vascular homeostasis by regulating endothelial barrier function, postnatal angiogenesis in mouse retina, inflammation, and vessel remodelling, as well as pathological angiogenesis and lymphangiogenesis in the mature tissues [2,3].

The Ang–Tie system comprises endothelial Tie1 and Tie2 (Tek) receptor tyrosine kinases (RTK) and the angiopoietin growth factor ligands of Tie2 (abbreviated as Angpt or Ang1, Ang2 and Ang4, the latter representing a human orthologue for mouse Ang3) [4–10]. The Tie receptors are almost exclusively expressed in the ECs of blood and lymphatic vessels. Tie1 is also expressed, albeit less, in certain haematopoietic cell lineages [11–13]. Tie2 expression is also evident in a subpopulation of type M2 Tie2-expressing monocytes (TEMs), in haematopoietic stem cells and in muscle satellite cells located among skeletal muscle myofibres in association with the microvasculature [14–17].

Angiopoietin-1 (Ang1) is an obligatory Tie2 agonist expressed in the developing myocardial wall and by mesenchymal cells surrounding the blood vessels [7]. In contrast with paracrine Ang1, Ang2 is expressed by ECs and acts as an autocrine context-dependent agonist/antagonist of Tie2 [8]. Ang2 is stored in ECs in intracellular secretory granules called Weibel–Palade bodies [18]. Inflammatory and hypoxic stimuli increase Ang2 expression, decreasing vascular stability and promoting endothelial activation, neoangiogenesis and remodelling [19–22]. In contrast, Ang1 promotes vessel stability (especially after angiogenic processes), inhibits tissue fibrosis, and mediates vessel normalization during anti-angiogenic therapy [23–25]. The orphan Tie1 receptor promotes tumour angiogenesis and atherosclerosis [26,27]. Activating mutations in the TIE2 receptor or the downstream signal transducer PIK3CA [p110α catalytic subunit of phosphatidylinositide-3 kinase (PI3K)] cause human venous malformations (VMs) [28–32] while loss-of-function TIE2 mutations result in glaucoma. Increased Ang2 levels are associated with numerous human diseases, including cancer, sepsis, infectious diseases, diabetes, atherosclerosis and tissue injury making the Ang–Tie system an attractive target for the development of future vascular therapies [3,33–35]. Here, we review recent findings highlighting the signalling properties of the Ang–Tie system, its function in vascular development and homeostasis, as well as its contribution to vascular pathologies.

## ANG–TIE SYSTEM IN CARDIOVASCULAR AND LYMPHATIC DEVELOPMENT

The initial VEGF-driven assembly of the primary blood vascular plexus that occurs by E9 (embryonic day 9) in mice is followed by VEGF-C-induced formation of the first lymphatic vessel structures between E10–11. Based on genetic deletions in mice, the angiopoietin ligands and the Tie receptors are required for cardiovascular and lymphatic development during mid-gestation, after the initial VEGF-induced signals (Table 1) [1].

**Table 1:** Genetic mouse models of the Ang–Tie pathway and associated vascular phenotypes

Gene	Gene modification	Phenotype	References
Tie1	Constitutive deletions (Tie1 lacZ/lacZ)	Death at E13.5-P0 depending on genetic background. Functional vasculature forms without defects up to E13.5. From E13.5 onwards haemorrhages observed throughout the body. Subcutaneous oedema and abnormal pattern of lymph sacs at E12.5. Death due to extensive haemorrhage embryonically or pulmonary oedema after birth.	[37,40,43]
Tie1	Hypomorphic allele with intronic neo cassette (Tie1 neo/neo) resulting in reduced expression of Tie1	Rarely lethal before E18.5, few survivors to adulthood. No haemorrhaging, no obvious defects in blood vessels. At E12.5 and onwards dilated jugular lymph sacs, severe generalized oedema, poorly functioning and disorganized lymphatic vessels in skin.	[42,43]
Tie1	Intracellular domain deletion (Tie1 ΔICD)	Subcutaneous edema by E13.5 and defective remodelling of the primary lymphatic network. In new-born mice conditionally induced Tie1 ΔICD resulted in defective formation of lymphatics at P7.	[45]
Tie1	Conditional deletion of Tie1fl/fl using inducible EC-specific Cdh5-Cre and Pdgfb-Cre ERT2 mouse lines	No apparent phenotype in vasculature when deleted in healthy adult mice. Reduced growth and angiogenesis of transplanted tumours. Deficient retinal angiogenesis when deletion induced from P1. Reduced vascular remodelling in response to recombinant or viral vector delivery of angiopoietins.	[27,65]
Tie1	Conditional deletion in lymphatic endothelium and developing valves using Nfatc1-Cre driver	Chylous ascites shortly after birth. Dilated intestinal lymphatic capillaries, agenesis of lymphatic valves, deficiency of collecting lymphatic vessels and poor lymphatic function.	[46]
Tie1	Heterozygous Tie1^+/−^ and inducible SCL-Cre ERT mediated deletion of Tie1fl/fl.	Decreased Tie1 expression led to attenuated inflammation and reduced number of atherosclerotic lesions in the aorta of adult apoE-deficient mice.	[26]
Tie2	Dominant-negative transgene and constitutive targeted deletion	Embryonic lethal at E9.5–10.5. Reduced growth of the myocardium with impaired trabecular organization. Defective vascular branching and remodelling, abnormally dilated vessels, defective patterning of small and large vessels.	[36,37]
Tie2	Conditional deletion using doxycycline inducible Tek2COIN × ROSA-rtTA Tet-On-Cre mice	Deletion in adult mice leads to buphthalmos phenotype due to defects in Schlemm's canal and lymphatic capillaries in corneal limbus by P21, progressing with age. Subcutaneous oedema and abnormal lymphatic patterning when deleted at E12.5.	[52]
Tie2	Conditional Tie2 deletion using inducible UBC-CreERT2 driver	Deletion of Tie2 in new born mice did not affect lymphatic vessel growth or maturation at P7.	[45]
Tie1 and Tie2	Constitutive double deletion	Lethal at E8.5–9.5. Similar defects as in Tie2 knockout mice, but more severe, defective cardiac development and vascular remodelling. Oedema and impaired vascular integrity similar to Tie1 knockout.	[156]
Tie1 and Tie2	Inducible EC deletion using Cdh5-Cre ERT2	More severe abnormalities in retinal vascular development in double deficient Tie1/Tie2 mice than in single null mice.	[54]
Ang1	Constitutive deletion	Death at E12.5. Growth retardation of heart, no trabeculae structure, collapsed and less complex endocardial lining similar to Tie2 knockout mice. In general less complex vasculature, primary vascular plexus remain primitive and large vessels are reduced in their number, branches and size. In ultrastructural analysis rounded ECs poorly associated with mural cells.	[38]
Ang1	Cardiomyocyte-specific deletion using Nkx2.5–Cre driver	Identical phenotype compared with constitutive Ang1 deletion.	[24]
Ang1	Cardiomyocyte-specific deletion using a-MHC-Cre driver	Ang1 deletion results in defective formation of the subepicardial coronary veins at E14.0, but not intramyocardial coronary arteries.	[39]
Ang1	Inducible ubiquitous Rosa-Cre deletion	Embryonic lethal when deleted before or at E12.5, due to cardiac defects and vascular abnormalities. Deletion after E12.5 has no clear effect on viability, fertility or phenotype of the mouse in unchallenged condition. Decrease in retinal vascularity at P5 and P17. Increased fibrosis in wound healing and severe kidney injury after streptozotocin-induced diabetes.	[24,55]
Ang1	Overexpression in skin under K14-promoter	Red skin with functionally intact vessels. Increased vessel number, with increased diameter and branching in the dermis in new born mice. The vessels were protected from inflammation-induced leakage.	[129,130]
Ang1	Expression in Ang2 locus	Rescues lymphatic developmental defects observed in Ang2 knockout mouse but not the abnormal remodelling of the vasculature in eye.	[47]
Ang2	Constitutive deletion	Depending on genetic background homozygous mice die within 2 weeks of birth or remain viable. Develop chylous ascites as a manifestation of poor lymphatic function. Architecture, vessel structure and valves are abnormal in the lymphatic system. Defective vascular remodelling in the eye and dysmorphogenesis of cortical peritubular capillaries in the kidney.	[47,48,157]
Ang2	Heterozygous deletion	Adult mice are protected from kidney and lung injury in murine sepsis, and from diabetes-induced pericyte dropout in the retina.	[119,136]
Ang2	Transgenic overexpression of Ang2 under Tie2 promoter element	Death at E9.5–10.5. Severe defects in cardiovascular development resembling phenotypes of Ang1 and Tie2 deficient mice suggesting a role as a natural Tie2 antagonist.	[8]
Ang2	Inducible human Ang2 overexpression using tetracycline regulated EC (Tie2) or cardiomyocyte (α-MHC) restricted activators	Hypotension, vascular hyperpermeability, cardiac hypertrophy and fibrosis, loss of pericytes.	[138]
Ang2	Inducible mouseAng2 overexpression in ECs using Cdh5 or Tie1-tTA/Tet-OS-Ang2 transgenic mouse lines	Increased tumour metastasis and decreased endothelial integrity. Remodelling of tracheal vasculature resulting in dilated capillaries.	[65,66,97]
Ang2	Inducible hAng2 overexpression in ECs using Tie1-tTA/Tet-OS transgenic lines	Impaired restoration of blood flow after arterial occlusion in the mouse limb.	[158]
Ang1 and Ang2	Conditional double knockout	Deletion at mid-gestation leads to similar phenotype as Tie2 knockout. Deletion at E12.5 results in subcutaneous oedema and abnormal patterning of dermal lymphatic vessels. Deletion at E16.5 shows no obvious phenotype until 21–28 days when high intraocular pressure, buphthalmos and features of glaucoma start to develop due to defects in Schlemm's canal and lymphatic capillaries in corneal limbus.	[52]

### Ang1/Tie2 in cardiovascular development

Constitutive deletions of *Tie2* and *Ang1* in mouse embryos result in embryonic lethality during embryonic day (E) 10.5–12.5, respectively, demonstrating their importance for embryonic vascular development [24,36–38]. Cardiac development was significantly impaired in the *Tie2* and *Ang1* deficient mouse embryos; only few myocardial trabeculations were present in the developing heart and the endocardial lining was retracted from the myocardial wall [24,36–38]. ECs of *Ang1* null embryos were found poorly associated with the underlying basement membrane and with periendothelial supporting cells [38]. Remodelling of the developing vasculature appeared compromised with less complex vasculature in *Ang1* and *Tie2* deficient embryos, with additional haemorrhaging and reduced number of ECs in embryos lacking Tie2 [36,38]. Somewhat surprisingly, Ang1 was dispensable after E12.5 in a mouse model allowing its conditional deletion, indicating that Ang1 is required during a specific developmental window [24]. Deletion of *Ang1* in cardiomyocytes largely phenocopied the vascular defects of the constitutive *Ang1* deletion allele, suggesting that the cardiac defects affected vascular development, possibly via hemodynamic effects [24]. More recently, cardiomyocyte-specific *Ang1* deletion was reported to also cause defective formation of the subepicardial coronary in the developing embryos [39].

### Tie1 in blood and lymphatic vascular development

The constitutive deletion of *Tie1* resulted in haemorrhages and death of mouse embryos by E13.5. Capillary remodelling and EC survival were compromised, but cardiac defects observed in the *Tie2* and *Ang1* deficient mouse embryos were absent from the *Tie1*-deficient embryos [40]. More detailed studies were performed using hypomorphic and tissue-specific inducible *Tie1* alleles and in a permissive genetic background where *Tie1*-deficient embryos lived longer (Table 1). These studies revealed that a reduced Tie1 protein level resulted in defective lymphatic development at E12.5 and onwards, after the formation of the first lymphatic structures in the jugular region [27,41]. An abnormal lymphatic phenotype, but no defects in the arterial or venous vasculature, was observed in hypomorphic mice expressing approximately 20% of normal Tie1 levels [42]. However, EC-specific *Tie1* deletion, with over 90% reduction in Tie1 protein, resulted in deficient retinal angiogenesis in postnatal mice [27], suggesting dose-dependent effects of Tie1 deficiency. It is noteworthy that Tie1 was not required under basal conditions in adult mice, but its deficiency resulted in decreased survival of ECs in the tumour vasculature [27].

The earliest lymphatic defects in *Tie1*-deleted mouse embryos included malformed jugular lymph sacs and lymphoedema [42,43]. The lymphatic vasculature develops centrifugally from the lymph sacs via sprouting of new lymphatic vessels from pre-existing ones. During development, the primitive lymphatic vessels are further remodelled into functionally-specialized initial and collecting lymphatics [44]. The remodelling of the primary lymphatic network to valve-containing collecting vessels was disrupted during both embryonic and postnatal development in mice homozygous for an allele lacking the Tie1 intracellular domain [45]. In addition, *Tie1* deletion in the developing lymphatic valves using the Nfatc1-Cre line prevented lymphatic valve specification of Prox1-positive lymphatic endothelial cells (LECs), and resulted in collecting lymphatic vessel deficiency [46]. In summary, these results highlight the important function of Tie1 in lymphatic vessel remodelling and valve morphogenesis.

### Ang2 and Ang1 in lymphatic development

The constitutive deletion of *Ang2* resulted in postnatal death of the *Ang2*-deficient pups in some mouse genetic backgrounds [47]. *Ang2*-deleted pups had a blood vascular phenotype, which was mainly restricted to the developing eye [47]. However, *Ang2* deficiency resulted in widespread lymphatic dysfunction, due to abnormal remodelling of the developing lymphatic vessels. Early lymphatic development occurred normally in *Ang2*-deficient embryos, but subsequent remodelling of the vessels resulted in narrowing of the lymphatic capillaries associated with abnormal vascular smooth muscle cell (SMC) coverage and defective lymphatic valve formation of collecting lymphatic vessels [45,47,48]. Administration of neutralizing Ang2 antibodies during embryonic development resulted in body swelling and haemorrhages starting at E12.5. At E18.5 the lymphatic vessels were hypoplastic due to reduced LEC proliferation and sprouting. Conversely, inducible Ang2 expression in ECs stimulated lymphatic hyperplasia in *Ang2* transgenic mouse embryos [49]. Marked changes were also detected in the LEC junctions in Ang2 antibody-treated and *Ang2*-deficient mouse embryos: Ang2 blocking inhibited the formation of specialized button-like junctions in lymphatic capillaries, instead they contained zipper-like junctions typically found in collecting lymphatic vessels [49]. Mechanistically, Ang2 blocking was found to interfere with VE-cadherin phosphorylation at tyrosine residue (Tyr) 685 in LECs, which has been reported to dynamically regulate vascular permeability [50,51]. Furthermore, collecting lymphatic vessels showed immature phenotype with poorly developed zipper-like junctions and lymphatic valves. Thus, Ang2 deficiency caused defective remodelling of both lymphatic capillaries and collecting vessels, thereby probably affecting both lymph uptake and delivery resulting in lymphoedema [47–49].

Interestingly, expression of *Ang1* in the *Ang2* genetic locus rescued the lymphatic abnormalities of *Ang2* deficient mice, suggesting that Ang1 and Ang2 have overlapping functions in the lymphatic vasculature [47]. In support of this, the lymphatic defects in mice, where both *Ang1* and *Ang2* were deleted, were more severe than in the *Ang2* knockout mice. The *Ang1/Ang2* double knockout mice were characterized by defective development of the Schlemm's canal, a specialized hybrid lymphatic vessel in the eye, and of lymphatic capillaries in the corneal limbus. These defects led to impaired drainage of aqueous humour, increased intraocular pressure (IOP) and glaucoma [52]. Furthermore, subcutaneous embryonic oedema was reported in the *Ang1/Ang2* double knockout, but not *Ang2* deleted embryos at E12.5, mimicking the embryonic conditional *Tie2* and *Tie1* deletions [43,52]. These reports suggest that Ang2, similarly to Ang1, functions as a Tie2 agonist during lymphatic development [52].

### Ang–Tie system in postnatal angiogenesis

The postnatal mouse retina provides a widely used model to investigate vascular sprouting and remodelling. Its highly organized vascular network is readily apparent in whole mount staining and the retina presents well-characterized, dynamic developmental stages in newborn mice [53]. The postnatal gene deletions of *Tie1*, *Tie1/Tie2, Ang1* and *Ang2* result in reduced growth of the vascular front from the optic nerve head during development of the superficial retinal vessels, indicating a role for the Ang–Tie system in sprouting angiogenesis of the retina.

Endothelial *Tie1* deletion decreased angiogenesis and increased Notch pathway activity in the retina, whereas pharmacological Notch suppression in the absence of *Tie1* promoted retinal hypervascularization, suggesting that Tie1 regulates vascular sprouting via the Notch pathway [27]. Additive inhibition of the retinal vascular front migration was observed when Ang2 blocking antibodies were administered to *Tie1*-deficient pups before postnatal day (P) 5 [27]. Similarly, retinal vascular development was more severely affected in the *Tie1/Tie2* double gene targeted pups, characterized by vascular tuft formation [54].

Ang1 has been reported to regulate both ECs and retinal astrocytes during retinal vascularization [55]. By activating endothelial Tie2 signalling in the developing blood vessels, Ang1 promotes vessel integrity. In addition, Ang1 interaction with αvβ5-integrin in the retinal astrocytes stimulates extracellular matrix (ECM) fibronectin (FN) production, thereby guiding the direction of endothelial tip cell migration and radial vascular front sprouting [55].

In the mouse eye, the development of retinal vasculature occurs simultaneously with the regression of embryonic hyaloid vessels in the primary vitreous body during the first postnatal weeks. In addition to the growth of the retinal vasculature, Ang2 is also required for regression of the hyaloid vessels at around P10 [47]. Interestingly, *Ang1* knockin in the *Ang2* genetic locus did not complement for Ang2 loss during regression of the hyaloid vasculature, suggesting that Ang2 specifically mediates vessel regression in this vascular bed [47]. This result resembles earlier studies where ectopic Ang2 expression in developing mouse embryos resulted in blood vessel regression, suggesting that Ang2 acted in an opposite fashion to the Ang1–Tie2 signalling axis [8].

## SIGNALLING MECHANISMS OF THE Ang–Tie SYSTEM

### Cell type and subcellular microenvironment- dependent Tie receptor signalling

Angiopoietins induce translocation and activation of the Tie receptors in certain subcellular compartments, which is dependent on the cell microenvironment, and may partly explain versatile functions of angiopoietins during vessel quiescence and remodelling [56–58]. In contacting ECs, such as those in the quiescent vasculature, angiopoietins induce the formation of Tie receptor signalling complexes *in trans* across the EC–EC junction. These junctional Tie complexes mediate cell survival signals via the PI3K-Akt pathway, which results in activation of the endothelial nitric oxide synthase (eNOS) [56,57,59]. Akt also phosphorylates the transcription factor forkhead box O1 (Foxo1), inducing its nuclear exclusion and reduced expression of Foxo1 target genes involved in metabolic and cell growth regulation [60]. Tie2 activation in EC junctions may inhibit endothelial permeability induced by inflammatory cytokines and growth factors. However, stimulation of overall Tie2 activity via inhibition of the vascular endothelial protein tyrosine phosphatase, VE-PTP, can also promote endothelial barrier function. This probably involves the small GTP binding protein Rap1 mediated regulation of Rac1 and stabilization of cortical actin cytoskeleton [61]. In mobile ECs, matrix-bound Ang1 activates Tie2 in EC-ECM adhesions, promoting extracellular-regulated kinases (Erk) and docking protein-2 (Dok2) activation, matrix adhesion and cell migration [56,57].

Of the three angiopoietins, Ang1 and Ang4 are obligatory Tie2 agonists, whereas the ability of Ang2 to induce Tie2 activation depends on a variety of contexts. Ang2 agonist activity has been reported in the lymphatic vascular bed, tumour vasculature and stressed ECs that have reduced Ang1–Tie2–Akt signalling, leading to feedback up-regulation of the Foxo1 target Ang2 [47,62–64]. In addition, in a transgenic mouse model, endothelial Ang2 expression stimulated Tie2 phosphorylation under baseline conditions, but inflammatory signals rendered Ang2 as an antagonist [65,66]. The exact mechanisms that regulate Ang2 agonist/antagonist function have not been fully elucidated, but they may depend on Ang2 multimerization or structural differences in the receptor-binding interface located in the C-terminal Tie2 binding fibrinogen-like domain of the angiopoietins [67]. Ang2 also induces Tie2 translocation to cell–cell junctions, but in this compartment activates Tie2 only weakly [57]. Since Ang1 and Ang2 bind in a similar fashion to Tie2 [67,68], Ang2 binding may lead to inhibition of Ang1-induced Tie2 signalling, especially when the Ang2/Ang1 ratio is elevated, such as in the diseased vasculature. Attenuation of the Ang1–Tie2 pathway can then lead to an increase in nuclear Foxo1, which stimulates Ang2 gene transcription [65,66]. Furthermore, Ang2, but not Ang1, induces Tie2 translocation into specific ECM contact sites that may weaken EC–ECM adhesion [58].

Tie1, the founder member of the Tie RTK family, remains as an orphan receptor with no identified ligand, despite overall homology with Tie2, especially in the intracellular tyrosine kinase domain [4]. However, via a direct molecular interaction with Tie2, Ang1 stimulates Tie1 phosphorylation with the same kinetics and doses, but more weakly than Tie2 phosphorylation in EC-EC junctions [54,65,69]. In adult mice, where Tie1 was deleted in ECs, angiopoietin induced vessel remodeling was decreased. In addition, Ang1 agonist activity was decreased, whereas that of Ang2 was lost in Tie1 deficient mice [65]. In the tip cells of angiogenic vessels, Tie1 can inhibit Tie2 surface presentation, thereby shaping the tip cell phenotype and Ang2 signalling [54]. Both Tie1 and Tie2 undergo proteolytic ectodomain cleavage in response to VEGF and inflammatory cytokines, although with different kinetics [70,71]. Interestingly, Tie1 was rapidly cleaved at the onset of acute inflammation in mice, whereas *Mycoplasma pulmonis* infection of mouse airways induced Tie1 cleavage over a period of several days correlating with loss of Ang2 agonist activity in the inflamed vasculature [65,66].

### Ang–Tie co-operation with integrins

**Figure 1:**
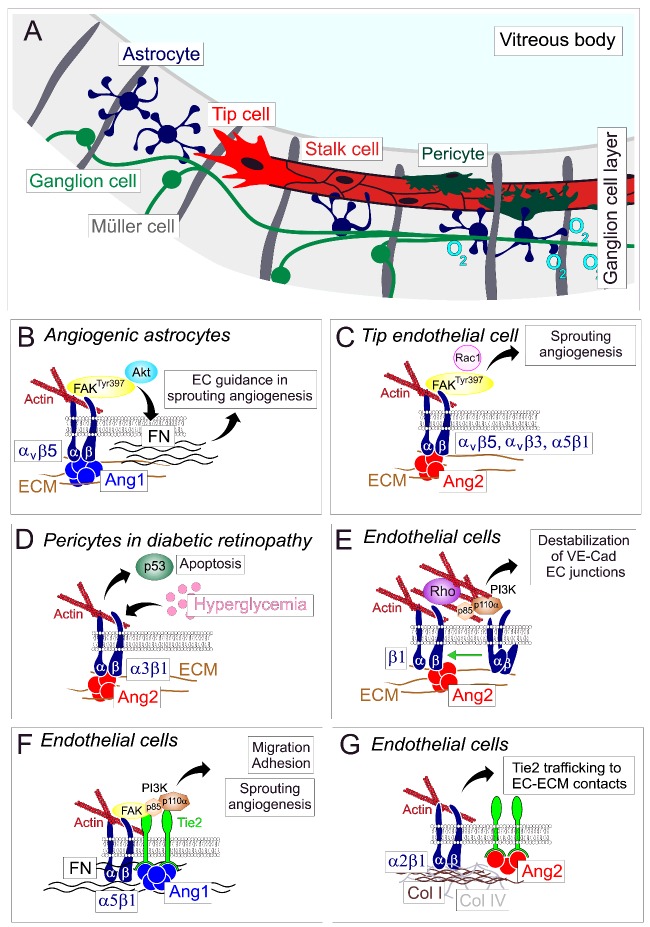
Ang–Tie signalling cross-talk with integrin cell adhesion receptors in the vasculature (**A**) Representation of superficial plexus (ganglion cell layer) of the developing mouse retina that provides a widely-used model for angiogenesis. Astrocytes migrate across the surface of the retina from the optic nerve in a manner that is dependent on signals from retinal ganglion cells [153]. Müller cells produce the majority of VEGF in the mouse retina [154]. Astrocyte template and a specialized endothelial tip cell guide the sprouting of new vessels, followed by lumen-forming stalk cells and pericytes, which stabilize newly formed vessels. (**B**) In Tie2-negative astrocytes Ang1 signals via integrin αvβ5 that activates fibronectin (FN) deposition via FAK and Akt pathways. This provides an extracellular scaffold for guided angiogenesis into the avascular retina in a mouse model for oxygen-induced retinopathy [55]. (**C**) Ang2 binds to αvβ3, αvβ5 and α5β1 integrins. In the tip cells of sprouting vessels that express a low level of Tie2, Ang2/integrin interaction induces FAK and Rac1 activation independent of Tie2 to stimulate sprouting angiogenesis [72]. (**D**) The expression of α3β1 integrin is increased in pericytes under hyperglycaemic conditions. In a mouse model for diabetic retinopathy, Ang2 induces apoptosis of pericytes via the p53 pathway that is attenuated by blocking integrin α3β1 [80]. (**E**) Direct binding of Ang2 activates α5β1-integrin (green arrow). In Tie2-silenced ECs Ang2 promotes the formation of β1-containing elongated matrix adhesions and actin stress fibres causing endothelial layer destabilization. This can be restored by inhibiting Ang2, β1-integrin, PI3 or Rho kinases and by expression of Tie2 ectodomain [75]. (**F**) Stable interaction between α5β1 integrin and Tie2 occurs in ECs plated on FN. This sensitizes Tie2 for low Ang1 concentration and recruits p85 and FAK to the α5β1/Tie2 complex. Ang1-induced Tie1 and Tie2 receptor interaction in EC-EC junctions, Tie receptor phosphorylation and Foxo1 inactivation as well as EC adhesion, migration and sprouting angiogenesis are dependent on α5β1 integrin [65,82,83]. (**G**) Ang-2 induced Tie2 translocation to the specific cell matrix contact sites is dependent on extracellular collagen types I and IV and their α2β1-integrin receptor, which regulates EC-ECM adhesion [58].

In addition to the Tie receptors, angiopoietins have been reported to interact with multiple integrin cell adhesion receptors both in ECs and non-ECs (Figure 1). Deposition of provisional ECM components (such as fibrin, FN, vitronectin and fibrillar interstitial collagen type I), as well as changes in the expression of integrin heterodimers serving as receptors for ECM ligands, regulate the EC–ECM interplay during angiogenesis and inflammation. These changes and the reported cross-talk of the Ang–Tie system with integrins may provide a layer of context-dependent regulation of angiopoietin signalling and angiopoietin-induced vascular responses.

Ang2 has been reported to stimulate focal adhesion kinase Fak phosphorylation on Tyr-397 via integrins in Tie2 low ECs and in tumour cells, thereby promoting cell migration [72,73]. As the endothelial tip cells of sprouting vessels express high levels of Ang2 and β1-integrin, but low levels of Tie2, the Ang2-integrin signalling axis may be well located to guide vessel sprouting during angiogenesis [72–74].

In endothelial monolayers, Tie2 silencing initiates an autocrine Ang2 signalling cascade, which stimulates the formation of actin stress fibres, leaky EC–EC junctions, and destabilization of endothelial monolayers. These changes on the cellular architecture were dependent on Ang2-induced translocation of the α5β1 integrin into the ends of actin fibres [75]. In addition, Ang2, but not Ang1, promoted the activation of α5β1-integrin, via the Ang2 N-terminus, which is distinct from the C-terminal fibrinogen-like domain mediating Tie2 binding [75]. Notably, decreased Tie2 expression is associated with infection- and inflammation-induced low blood flow *in vivo* that may promote endothelial monolayer destabilization in the diseased vasculature [76,77].

Ang2 is known to promote pericyte dropout in diabetic mouse retina, associated with loss of integrity of the retinal vasculature, and consequently, vascular leak, ischaemia and neovascularization, causing vision loss [78,79]. Recently, Ang2 was found to induce pericyte apoptosis via α3β1-integrin under high glucose conditions [80]. Furthermore, the Ang2–Tie2 axis stimulated proteasome degradation of the vascular stabilizing αvβ3 integrin [81], and decreased EC-ECM adhesion by inducing Tie2 translocation to specific EC-ECM contact sites in an α2β1-integrin dependent manner [58]. Collectively, these results suggest that the Ang2-integrin signalling promotes endothelial destabilization and angiogenesis.

As mentioned earlier, Ang1 stimulates FN production via astrocyte-expressed αvβ5-integrin, thereby guiding directed EC migration in the developing retinal vasculature [55]. In ECs, α5β1-integrin was required for Ang1 induced formation of a Tie1-Tie2 receptor complex in EC-EC junctions, Tie2 phosphorylation and downstream Foxo1 phosphorylation [65]. In addition, FN was reported to sensitize ECs for low levels of Ang1 and promote Tie receptor interactions with α5β1-integrin [82,83], whereas venous malformation-causing activating mutations in TIE2 and PIK3CA resulted in loss of FN production by ECs [29,30]. In summary, these results indicate that Ang1, Ang2 and Tie2 control the EC-ECM interplay together with the integrins, which may also serve as receptors for angiopoietins in non-ECs or in ECs with low Tie2 levels. Future studies should elucidate the involvement of the angiopoietin-integrin signalling axis in vascular processes *in vivo*.

### VE-PTP regulation of the Ang–Tie signalling

**Figure 2:**
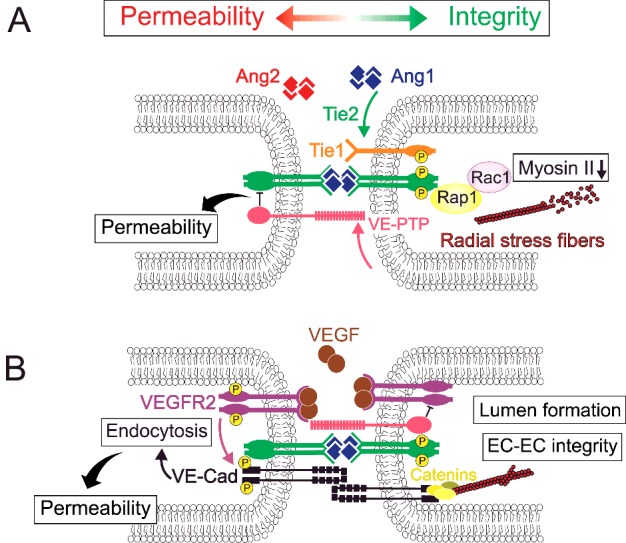
VE-PTP regulation of junction integrity and lumen formation via Tie2, VE-cadherin and VEGFR2 Molecular mechanisms during destabilized and stabilized endothelium are depicted on left and right, respectively. (**A**) Multimeric Ang1 assembles a junctional signalling complex formed by Tie2 receptors from opposing cells [56,57]. This complex also contains Tie1 and is dependent on α5β1 integrin [57,65]. Ang1-Tie2 can also recruit VE-PTP into EC–EC contacts [57]. VE-PTP dephosphorylation of Tie2 promotes vascular permeability. Activated Tie2 stimulates Rap1 GTPase, which reduces radial stress fibres via Rac1 and nonmuscle myosin II, independent of VE-cadherin [61]. Increase in Ang2 can antagonize vascular stabilizing Ang1–Tie2 signalling in EC–EC contacts [57]. (**B**) Left, activated VEGFR2 induces a signalling cascade leading to phosphorylation of the VE-cadherin, resulting in disassembly of EC–EC junctions [155]. Right, at EC–EC junctions, VE-PTP associates with and indirectly dephosphorylates VEGFR2 via a Tie2-dependent mechanism [92,93]. This down-regulates VE-cadherin tyrosine phosphorylation, and promotes EC polarity and lumen formation.

VE-PTP (also known as HPTPβ, PTPRB, RPTPβ) is an EC-specific protein tyrosine phosphatase that regulates Ang–Tie signalling (Figure 2). VE-PTP is indispensable during mouse vascular development, as its constitutive deletion results in incomplete angiogenesis and embryonic death at around E11 [84]. VE-PTP associates with Tie2, and negatively regulates Tie2 signalling via Tie2 dephosphorylation [85]. In embryonic allantois tissue and in newborn mice, anti-VE-PTP antibodies induced increased Tie2 and Erk1/2 activation, EC proliferation and vessel enlargement. These changes demonstrated that VE-PTP controls blood vessel development and vessel size at least partially via Tie2 [85].

VE-PTP also associates with VE-cadherin, which supports EC–EC adhesion and endothelial junction integrity [86–88]. Vascular permeability and leukocyte transmigration during inflammation are associated with phosphorylation of specific tyrosine residues in the cytoplasmic tail of VE-cadherin, and require VE-PTP dissociation from VE-cadherin [51,89,90]. The *VE-PTP* gene promoter contains a HIF2α target sequence, which responds to hypoxia via increased VE-PTP expression [91]. Interestingly, pharmacological activation of HIF2α, using a PHD2 inhibitor, increased the integrity of adherence junctions, and prevented pulmonary oedema and neutrophil transmigration in a lipopolysaccharide (LPS)-induced murine systemic inflammation model [91].

A recent study demonstrated that VE-PTP blocking antibodies, a VE-PTP targeted pharmacological inhibitor (AKB-9778), and *VE-PTP* gene deletion inhibited vascular leak and leukocyte endothelial transmigration during inflammatory stimulus [61]. Inhibition of VE-PTP activity resulted in activation of Rap1-Rac1 signalling and stabilization of the endothelial cortical actin cytoskeleton via Tie2, but independently of VE-cadherin [61]. However, in a *Tie2*-deficient mouse background, VE-PTP inhibition promoted vascular leak via VE-cadherin, suggesting a model where inhibition of VE-PTP improves endothelial barrier function via increased Tie2 activation, whereas in the absence of Tie2, VE-PTP inhibition increases permeability, via increased VE-cadherin phosphorylation and internalization [61].

Ang1 stimulation of ECs promotes the recruitment of VE-PTP into EC–EC junctions, where VE-PTP is also known to associate with VEGFR2 and VEGFR3, in addition to Tie2 and VE-cadherin [57,92]. As VE-PTP also dephosphorylates VEGFR2, the Ang1-induced VE-PTP recruitment to the EC–EC junctions may result in down-regulation of VEGFR2 signalling, thereby contributing to improved barrier function [93]. In mouse embryoid bodies and in the developing zebrafish vasculature, VE-PTP deficiency resulted in defective EC polarization and lumen formation by stalk cells of newly-formed vascular sprouts, an effect probably mediated via increased VE-cadherin phosphorylation and VEGFR2 activity [93].

## Ang–Tie SYSTEM IN TUMOUR ANGIOGENESIS AND METASTASIS

In addition to its role in postnatal angiogenesis in the developing mouse retina, the Ang–Tie system is involved in tumour angiogenesis. Ang2 blocking biologicals and the genetic deletion of *Tie1* decreased tumour angiogenesis and tumour growth by reducing tumour cell proliferation and EC sprouting and by inducing vessel regression and EC apoptosis [27,94]. Ang2 blocking also promoted a normalized tumour vascular phenotype with increased pericyte coverage of tumour vessels [94,95]. At sites of distant lung metastasis, Ang2 blocking antibodies improved EC–EC junctions and regulated proangiogenic TEMs of the tumour stroma, thereby decreasing metastatic dissemination and growth [96–97].

Anti-Ang2 monoclonal antibodies and peptide-Fc fusion proteins that selectively neutralize the interaction between Ang2 or both Ang1 and Ang2 with Tie2 have demonstrated inhibition of tumour growth and angiogenesis in human tumour xenografts and orthotopic mouse tumours. This inhibition is elicited via reduced EC proliferation, decreased vessel sprouting, induction of necrosis and improved pericyte-endothelial interactions, as well as via vessel regression in certain tumour models [20,34,94–99]. In general, VEGF blocking antibodies or the VEGF-trap increase the tumour growth inhibition obtained via Ang2 blocking antibodies [64,94,98,101]. In certain tumours, resistance to VEGF-targeted therapies has been associated with therapy-induced Ang2 expression, and in these tumours the combination with Ang2 inhibition has been reported to overcome such resistance [102,103]. Conversely, Ang1 blocking failed to provide tumour growth inhibition, but it prevented tumour vessel normalization including improved EC–EC contacts and EC–pericyte interactions, induced by Ang2 blocking peptibodies, suggesting that Ang1 contributes to vessel stabilization during anti-angiogenic therapy [95,99,104]. The Ang2-targeted investigational drugs are currently tested in clinical oncology trials [34].

Tie1 is expressed in the vasculature of human tumours and conditional *Tie* deletion was demonstrated to inhibit tumour growth and neovascularization [27,105]. Tumours grown in *Tie1*-deficient mice were smaller and contained less blood vessels when compared with tumours grown in wild-type mice [27]. A closer analysis of the tumours in the *Tie1*-deleted mice revealed less vascular sprouts, increased EC and tumour cell apoptosis, decreased vascular perfusion and association of degenerating ECs with small intravascular fibrin deposits in the tumour vasculature [27]. Tumour growth was further reduced by the soluble Tie2 ectodomain, but anti-VEGF therapy in the *Tie1*-deleted mice [27]. Importantly, the normal vasculature was not affected by *Tie1* deletion, suggesting that Tie1 deletion is detrimental in angiogenic, but not quiescent ECs [27].

### Ang–Tie system in metastasis

Ang2 blocking inhibits tumour metastasis, potentially via multiple mechanisms. Tumour-associated lymphangiogenesis is associated with lymph node metastasis [106], and Ang2 blocking antibodies reduced the number of lymphatic vessels and lymph node metastasis of human tumour xenografts in mice [97]. During haematogenous metastasis, the vascular endothelium forms a barrier for tumour cells extravasating from the blood circulation at sites of distant metastasis [107]. Ang2 blocking decreased tumour cell homing to the lungs from the blood circulation by improving EC–EC junctions of pulmonary capillaries as observed using transmission electron microscopy [97]. In line with this result, Ang2 blocking antibodies inhibited tumour cell transendothelial migration *in vitro* [75]. In addition, Ang2 blocking targeted the Tie2 positive pro-angiogenic macrophage (TEM) population in the lung metastasis. Ang2 blocking decreased the up-regulation of Tie2 in tumour TEMs, which is required for their association with tumour blood vessels and proangiogenic activity [96]. In another study, the combination of Ang2 blocking with low-dose metronomic chemotherapy was found to inhibit metastatic growth, via attenuation of inflammatory and angiogenic endothelial responses, resulting in decreased recruitment of tumour-promoting CCR2^+^Tie2^−^ metastasis-associated macrophages and pro-tumorigenic bone marrow-derived myeloid cells [108].

In comparison with Ang2 blocking reagents, constitutive *Ang2* gene deletion has only a minor effect on tumour growth, but results in tumour vessel phenotype characterized by narrow vessel diameter and higher pericyte coverage [109]. Interestingly, metastatic growth of colon adenocarcinoma cells in the lungs was decreased in *Ang2* deficient mice, whereas liver metastasis was increased upon deletion of the *Ang2* gene [110]. These results imply organ specific differences in the regulation of metastasis progression via Ang2. One possible mechanism for increased liver metastasis in the Ang2 gene targeted mice involves tissue or metastatic-niche-specific up-regulation of VEGF-independent compensatory angiogenic pathways, such as the granulocyte-colony stimulating factor (G-CSF) and CXCL1 [110]. The mechanism of lung metastasis formation by melanoma, lung adenocarcinoma and renal cell carcinoma cells was found to involve tumour-derived VEGF, and VEGF-dependent activation of the calcineurin-NFAT pathway in the metastatic niche. NFATc1 binds to the *Ang2* gene region, thereby increasing *Ang2* gene transcription in the metastatic microenvironment [111].

## Ang–Tie SYSTEM IN VASCULAR DISEASES

### Ang–Tie system in ocular neovascularization

Vascular development in the eye includes both regression of embryonic hyaloid vessels in the vitreous and around the lens, and the simultaneous formation of a vasculature in the retina. This change in the ocular vasculature takes place during the first postnatal weeks in mice, and is dependent on the Ang–Tie signalling system, as discussed above.

Studies using transgenic mouse models have elucidated the functions of angiopoietins in VEGF-induced retinal vascularization. Adenoviral VEGF expression induced sprouting of the superficial capillaries only when co-expressed with Ang2, whereas VEGF was sufficient to stimulate sprouting of the deep retinal capillary bed, due to continuous Ang2 expression in these vessels after P7 in mice [112]. Inducible ubiquitous or photoreceptor restricted expression of Ang2 or VEGF demonstrated that Ang2 stimulated retinal neovascularization (NV) in concert with VEGF, but induced vessel regression when VEGF level was low [113]. In contrast, transgenic expression of Ang1 prevented VEGF-induced NV and exudative retinal detachment in VEGF/tet/opsin mice, but did not affect established retinal and choroidal NV [114–116]. Thus, ectopic Ang1 makes ECs less responsive to VEGF-induced signals, whereas the sensitivity of ocular vasculature to Ang2 is determined by the VEGF/Ang2 ratio and additional factors, which may include integrins and ECM components [117]. Furthermore, conditional *Ang1* gene deletion aggravated, whereas VE-PTP inhibition via administration of AKB-9778 attenuated, both choroidal and retinal pathological NV, following laser-induced rupture of the Bruch's membrane and oxygen-induced retinopathy in mice, respectively [55,116,118].

Retinopathy is a common complication of diabetes, and is associated with pericyte loss, which is driven by Ang2, potentially via an integrin-dependent mechanism in high glucose environments [78,80]. Diabetic vascular dysfunction also affects various other organs and tissues in extremities, heart, nerves, wounds and kidneys. The contribution of the Ang–Tie system to the pathogenesis of ocular and diabetes-associated vascular diseases is a field of intense research [117,120].

### Angiopoietin ligand cooperation in the development of Schlemm's canal and in the lymphatic drainage of the eye

In the eye, aqueous humour fills the space between the cornea and lens, regulating the homeostasis of anterior ocular tissues. Aqueous humour is continually produced by the ciliary epithelium, and to maintain physiological IOP, the rate of drainage has to be balanced. In the conventional outflow pathway, aqueous humour is drained via the trabecular meshwork into Schlemm's canal (SC), and to the systemic circulation. The increased IOP can be caused by dysfunctional SC and is a common cause of glaucoma that can result in optic nerve damage and visual field loss. SC is a specialized vascular structure, showing similarities with the blood and lymphatic vascular system. Using conditional gene deletions in mouse models, cellular and molecular mechanisms for SC development have been recently discovered [52,121,122]. SC originates from the venous vasculature during mouse postnatal development [121]. In this process, aqueous humour outflow up-regulates the expression of the lymphangiogenic transcription factor PROX1, which is also important to maintain SC functionality [121,122]. SC then acquires many lymphatic characteristics (such as expression of VEGFR3, CCL21 and Itga9) and, unlike the blood vessels, contains no pericytes or SMCs. However, mature SC did not express all markers of differentiated lymphatic vessels (lack of luminal valves and podoplanin expression, low LYVE-1) suggesting that SC represents a unique, lymphatic-like vessel type. Previous studies have suggested that Ang1 and Ang2 function as agonistic ligands in lymphatic vasculature [47]. Double *Ang1*/*Ang2* gene deletion and embryonic *Tie2* deletion at E16.5 led to defective development of the SC and lymphatic capillaries in corneal limbus, resulting in clinical features of glaucoma (high IOP, buphthalmos, retinal ganglion degeneration and vision loss) 21–28 days after birth [52]. The identification of TIE2 mutations underlying human primary congenital glaucoma, an important cause of childhood blindness worldwide, demonstrated that the Ang-Tie pathway contributes to the pathogenesis of glaucoma also in human patients [123].

### Ang–Tie system in inflammation-induced vascular permeability and remodelling

Ang1 inhibits paracellular permeability induced by several inflammatory cytokines and growth factors, such as histamine, thrombin and VEGF [23,124], whereas Ang2 acts in synergy with inflammatory cytokines to promote vascular leak [21], although in at least one study the opposite was observed [62]. Tie2 appears to be important for vascular barrier function, as Tie2 silencing *in vivo* increased both basal and LPS-induced vascular permeability in the lungs [61]. In addition, low Tie receptor expression predisposed mice for murine Ebola virus-induced haemorrhagic fever [125]. Ang1, via Tie2, stimulates the activity of several downstream signalling pathways, which stabilize VE-cadherin in EC–EC junctions and maintain the cortical actin cytoskeleton [3,61,126]. The latter mechanism may be especially important in the brain and skin vasculatures, as VE-cadherin (*Cdh5*) gene-deficient mice demonstrated that VE-cadherin was not required in these tissues for the maintenance of the vascular barrier under basal conditions [61]. Ang1 can also directly modify basal microvessel permeability by increasing the endothelial glycocalyx formation [127].

During chronic inflammation, capillary-to-venous remodelling expands the vascular area, allowing plasma leakage and leukocyte emigration. Chronic *Mycoplasma pulmonis* infection in mouse airways results in increased Ang2 and loss of phosphorylated Tie2 from the tracheal blood vessel endothelium [128]. Specific neutralization of Ang2 using function blocking antibodies decreased vessel remodelling, vascular leak, and leucocyte influx, and these effects were further enhanced by synergistic neutralization of tumour necrosis factor (TNF)-α [22,128]. Ectopic expression of Ang1 also stimulates vascular remodelling, including the production of larger, more numerous, and more highly branched vessels in the skin of transgenic mouse embryos [129,130]. In addition, adenoviral vector delivery of Ang1 induces capillary-to-venous remodelling in the trachea producing non-leaky enlarged vessels, with enhanced blood flow in adult mice [131,132]. Notably, the Ang1-induced vessels are non-leaky and have increased pericyte coating in comparison with VEGF-induced vessels in the mouse skin [129,130]. Ectopic administration of COMP-Ang1, a recombinant Ang1 protein, during chronic *M. pulmonis* infection prevented vascular leak, and these anti-permeability effects of Ang1 required platelet derived growth factor (PDGF)-dependent pericyte functions [133]. In addition, adenoviral vector-mediated delivery of Ang1 in mice during postnatal life induced EC proliferation in the absence of angiogenic sprouting, resulting in vessel enlargement [134].

In diseases characterized by endothelial activation and compromised vascular function, the circulating Ang2 levels are increased beyond those of Ang1, thereby probably shifting Ang1–Tie2 signalling towards Ang2 [33]. The increase in Ang2 gene transcription may involve the Foxo1 transcription factor, which binds the Ang2 gene promoter in ECs under stress, where Ang1 levels are already decreasing and P-Akt is low [62,135]. In such cells, Ang2 may initially protect the endothelium, however, as shown by very recent reports, the Ang2 agonist activity is lost in inflammation, decreasing Tie2 phosphorylation and resulting in vessel destabilization [65,66]. Indeed, mice with reduced Ang2 gene dosage developed milder kidney and lung injury in mouse models of sepsis [136]. Furthermore, anti-Ang2 neutralizing antibodies and administration of recombinant Ang1 alleviated vascular complications in murine sepsis and acute inflammation [137,138], and protected rat cardiac allografts from endothelial injury and inflammatory responses [139]. Ang2 has also been identified as an endothelial-derived signal to control tissue regeneration in the liver, by regulating both hepatocyte proliferation and vascularization after partial hepatectomy [140].

### Role of Ang–Tie system in shear stress responses and atherosclerosis

Atherosclerosis is a chronic inflammatory disease of the arteries characterized by accumulation of inflammatory cells and subendothelial lipids, resulting in the formation of atherosclerotic plaques that may lead to rupture or occlusion of arteries. Atherosclerotic lesions develop in specific regions in the arteries; predominantly at bifurcations, the inner curvature and branches of the aorta, where non-laminar, disturbed blood flow causes low or oscillatory shear stress to the endothelium. Non-laminar flow promotes chronic endothelial inflammation, which is an important risk factor for the formation and progression of the atherosclerosis plaques. In contrast with disturbed flow, laminar flow and high shear stress protect against atherosclerosis [141].

Tie1 expression is modulated by haemodynamic forces; nonlaminar flow induces Tie1 expression in atherogenic vascular niches [26,142], whereas laminar flow in cultured ECs down-regulates Tie1 expression [26,143]. The function of Tie1 in atherosclerosis was investigated in *apoE-*deficient mice using the conditional *Tie1* allele. Decrease in Tie1 expression in this model led to a dose-dependent reduction in atherosclerotic lesions in the distal aorta, associated with increased eNOS and IkBα, and decreased ICAM-1 expression in ECs under laminar shear stress. These results indicate a proinflammatory disease-promoting function for Tie1 in atheroma formation [26].

In addition to Tie1, laminar shear stimulates a polarized subcellular distribution of VE-PTP [144], and down-regulates Ang2 expression via KLF2 transcription-factor mediated miRNA regulation [145,146]. *In vivo* Ang2 is expressed in the atherosclerosis-prone region of the lesser curvature of the aortic arch, which experiences disturbed flow pattern [146]. Studies using animal models have produced conflicting results, however, and the exact role of Ang2 for atherosclerosis development remains to be elucidated [147,148].

### Activating mutations of the TIE2-PIK3CA pathway in venous malformations

VMs represent the most common defects of vascular morphogenesis, characterized by localized, abnormally enlarged, venous-like channels. Depending on the size and anatomical location, VMs can cause severe disfigurement, pain, and obstruction. The genetic cause of most VMs has been revealed; more than 50% of VMs are positive for activating mutations in *TIE2* gene [149] and a significant proportion of TIE2 mutation-negative VMs carry mutations in *PIK3CA*, which encodes the p110α catalytic subunit of PI3K [29]. When expressed as mutant proteins in cultured ECs, recurrent mutations in TIE2 and PIK3CA resulted in the same cellular abnormalities, which were restored by the p110α-specific inhibitor [29]. In addition, the TIE2 and PIK3CA mutations resulted in the formation of VM lesions when mutant genes were expressed by transplanted ECs in mice [30,150], and in a genetic mouse model [31,32], respectively. These results indicate that the TIE2 receptor and the PIK3CA signal transducer participate in the same VM signalling pathway, opening new possibilities for evidence-based molecular therapies against VMs [29,31,32,150]. Recent genetic analysis have revealed that allelic *TIE2* double mutations are enriched in blue rubber bleb nevous syndrome (BRBN), characterized by multiple VM lesions [151]. ECs expressing recurrent BRBN TIE2 mutation showed increased colony forming capacity *in vitro* assays that may contribute to the clinically distinct multifocal VM phenotype in BRBN patients [151].

Abnormal cellular signalling induced by 22 VM associated TIE2 mutations was recently investigated using cultured ECs, tissue samples from a VM mouse model, and patient biopsies [30]. The TIE2 mutant cell phenotypes included defective TIE2 trafficking, loss of endothelial cobblestone organization due to MAP kinase-dependent FN deficiency, increased Akt and Erk1/2 phosphorylation and aberrant organization of perivascular ECM. In addition to these altered cellular processes, the plasminogen/plasmin proteolytic pathway was up-regulated, probably accounting for high fibrin D-dimer levels, a major feature of unknown cause distinguishing VMs from other vascular anomalies [30]. In addition, gene expression profiling of mutant TIE2 expressing ECs revealed an Akt-Foxo1 dependent decrease in the expression of PDGFB, which may explain the characteristic irregular SMC coverage of VM lesions [152].

## CONCLUSIONS

The Ang–Tie system, which was first identified approximately 25 years ago, regulates both cardiovascular and lymphatic development, vascular homeostasis, and pathological inflammatory and angiogenic responses, including tumour angiogenesis. Ang2 is up-regulated in numerous human diseases, and Tie1 also promotes pro-inflammatory and pro-angiogenic signals in atherosclerosis and in tumours, respectively. The vascular stabilizing function of the Ang1–Tie2 pathway is compromised in disease, via down-regulation of this ligand–receptor system and up-regulation of Ang2. Because of the unique vascular stabilizing and destabilizing functions mediated by Ang1 and Ang2, respectively, growing interest is focused on the Ang–Tie system in inflammatory and neovascular diseases, associated with vascular leak and endothelial dysfunction. Future work is expected to reveal if targeting the Ang–Tie signalling system can provide benefit when used in combination with VEGF inhibitors or in vascular diseases where VEGF signalling inhibitors are not primarily exploited.

## Abbreviations

E: Embryonic day
Ang: angiopoietin
EC: endothelial cell
ECM: extracellular matrix
Erk: extracellular signal-regulated kinases
FN: fibronectin
Foxo1: forkhead box O1
IOP: intraocular pressure
LEC: lymphatic endothelial cell
P: postnatal day
PDGF: platelet derived growth factor
PI3K: phosphatidylinositide-3 kinase
RTK: receptor tyrosine kinase
SC: Schlemm's canal
SMC: smooth muscle cell
TEM: Tie2 expressing monocyte
VEGF: vascular endothelial growth factor
VEGFR: vascular endothelial growth factor receptor
VM: venous malformations
